# Development and Validation of a Gene-Targeted dCAPS Marker for Marker-Assisted Selection of Low-Alkaloid Content in Seeds of Narrow-Leafed Lupin (*Lupinus angustifolius* L.)

**DOI:** 10.3390/genes10060428

**Published:** 2019-06-04

**Authors:** Magdalena Kroc, Katarzyna Czepiel, Paulina Wilczura, Monika Mokrzycka, Wojciech Święcicki

**Affiliations:** 1 Department of Genomics, Institute of Plant Genetics, Polish Academy of Sciences, Strzeszyńska 34, 60-479 Poznań, Poland; kcze@igr.poznan.pl (K.C.); mwil@igr.poznan.pl (P.W.); wswi@igr.poznan.pl (W.Ś.); 2 Department of Biometry and Bioinformatics, Institute of Plant Genetics, Polish Academy of Sciences, Strzeszyńska 34, 60-479 Poznań, Poland; mmok@igr.poznan.pl

**Keywords:** narrow-leafed lupin, alkaloids, dCAPS marker, low-alkaloid, marker-assisted selection, single-nucleotide polymorphism, domestication traits, seed quality

## Abstract

Low-alkaloid content is an important breeding target to improve the quality of lupin seeds. An APETALA2/ethylene response transcription factor, *RAP2-7*, is likely a candidate gene for the major alkaloid locus *iucundus*, and plays a crucial role in regulation of seed alkaloid content in narrow-leafed lupin (NLL; *Lupinus angustifolius* L.). Here, we exploited a single-nucleotide polymorphism within *RAP2-7* credibly associated with seed alkaloid content, to develop the co-dominant derived cleaved amplified polymorphic sequence (dCAPS) marker iuc_RAP2-7. Marker validation in 202 NLL accessions demonstrated that seed alkaloid content ≥0.9% of the seed dry weight was associated with the high-alkaloid marker band (*Iucundus* genotypes), whereas alkaloid content up to 0.5% of the seed dry weight was associated with the low-alkaloid marker band (i*ucundus* genotypes). Within a given detection limit, iuc_RAP2-7 unambiguously identified all but three low-alkaloid accessions. The latter accessions apparently have a different regulatory mechanism for seed alkaloid content because the *RAP2-7* gene/putative promoter sequence and expression of alkaloid-associated genes in the leaves of the three ambiguous accessions were similar to those of bitter *Iucundus* lines. We consider the iuc_RAP2-7 marker is a powerful tool that will facilitate NLL marker-assisted selection by rapid rejection of bitter *Iucundus* genotypes and thus accelerate development of new low-alkaloid cultivars.

## 1. Introduction

Lupin seeds accumulate lysine-derived quinolizidine alkaloids (QAs) [1]. Owing to health constraints arising from their toxicity to humans and animals, the QA content in seeds limits the use of lupins as food and feed. Therefore, the low alkaloid content is considered to be one of the pivotal domestication traits, affecting lupin seed quality. Currently, the accepted industry threshold is 0.02% of the seed dry weight (SDW) and lowering the alkaloid content remains an important objective in lupin breeding [2,3,4]. The biosynthesis pathway and accumulation of QAs in lupins remains poorly understood and, to date, the narrow-leafed lupin (NLL; *Lupinus angustifolius* L.) has been the most extensively investigated in this regard [5,6,7,8,9,10,11,12,13]. Several single recessive genes arising from natural mutation, which confer reduced alkaloid content in the seeds, have been described for NLL [14,15]. Among these genes, the *iucundus* allele has been the most widely used in breeding and is considered to be a major gene regulating seed alkaloid content [16].

Development of new cultivars that incorporate several desirable agronomic traits by means of conventional breeding techniques is a laborious and time-consuming process, which might be greatly accelerated by the implementation of genomic tools [17,18]. Molecular markers employed in genomics-assisted breeding are increasingly successfully applied in choosing parental lines in crosses as well as selection of germplasm with desirable characteristics at early stages of breeding programs. Therefore, by providing improved precision and efficiency in material selection, the markers significantly accelerate development of new cultivars [17,18,19]. The recent, rapid growth of transcriptomics and genomics data has facilitated the identification of candidate genes associated with important agronomic traits among many plant species, including legumes [13,17,18]. Such data provide the opportunity to develop molecular markers targeting a specific gene region (gene-targeted markers), and in a more advanced approach, functional markers derived from polymorphic sites causally affecting phenotypic trait variation [20,21,22,23]. The utility of gene-targeted and functional markers far exceeds the effectiveness of less advanced classes of random, non-targeted markers developed without prior sequence information, or knowledge of their function and their position in relation to a gene of interest [21,23].

The use of molecular markers associated with QA content in NLL has been described previously by Li et al. [16]. The authors developed the microsatellite-anchored fragment length polymorphism-derived PCR marker lucLi linked to the low-alkaloid locus *iucundus* (0.9 cM). Although further estimation of the lucLi marker showed perfect correlation between the marker genotype and phenotype in the case of 25 NLL cultivars, its accuracy in the case of 125 core-collection accessions was assessed to be 86.4%. The disagreement between marker genotype and phenotype is a natural consequence of genetic recombination between the marker and the targeted gene, which may occur if a marker is not anchored into the gene sequence. As a result, the usefulness of the established iucLi marker for marker-assisted selection (MAS) in NLL breeding is limited. Similar results were obtained when application of a sequence-specific PCR marker PauperM1, closely linked (1.4 cM) to the low-alkaloid locus *pauper* in white lupin (*Lupinus albus* L.), was verified on germplasm from Australian lupin breeding programs [24]. 

Identification of a gene underlying the trait of interest is a critical step affecting MAS robustness [17]. In our previous studies based on comparative transcriptomic analyses of bitter (high-alkaloid, *Iucundus*) and sweet (low-alkaloid, *iucundus*) accessions of NLL, we identified an APETALA2/ethylene response transcription factor (AP2/ERF TF), *RAP2-7*. *RAP2-7* co-segregated with the *iucundus* locus and was located within a region containing highly significant QTLs that affect QA composition (linkage group NLL-07). Therefore *RAP2-7* is likely a vital regulatory gene of alkaloid biosynthesis/accumulation in NLL [13]. In this study, we report the development of a user-friendly PCR marker for the *iucundus* candidate gene allele. This newly developed molecular marker has been validated in NLL genotypes representing different classes of origin (wild accessions, cultivars, and other man-made accessions). Described here marker is a useful potential tool for exploration of MAS for low-alkaloid content in NLL.

## 2. Materials and Methods

### 2.1. Plant Material for Marker Development and Validation

Seeds of the 202 NLL accessions deposited in the Polish *Lupinus* Gene Bank were provided by Poznan Plant Breeders Ltd., Wiatrowo Branch, (Wiatrowo, Poland) (Supplementary Table S1). The accessions were maintained as pure lines and encompass different classes of origin, namely:

- CO—wild and primitive populations originating from places of natural distribution—60 accessions;
- LR—landraces—two accessions;
- XD—breeding lines (created by man as an effect of selection after crossings or induced mutations (excluding cultivars))—57 accessions;
- CV—past and present registered cultivars—76 accessions;
- unknown classification—seven accessions.

Seeds of the 83A:476 (domesticated) and P27255 (wild) accessions (the parental lines of the NLL mapping population) were provided by the Department of Agriculture and Food, Western Australia (DAFWA), Australia. 

The afore-mentioned seeds were grown in field experiments at Wiatrowo, Poznan Plant Breeders Ltd., Poland.

To confirm marker evaluation results and seed alkaloid profiles, the seeds of two NLL accessions, Brianskij-35 (95826) and Brianskij-123 (95927), were obtained from the National Centre for Plant Genetic Resources: Polish Genebank, Radzików, Poland and were grown in a greenhouse. 

For DNA and RNA extraction, young leaves were collected and frozen at −80 °C. The leaves for RNA extraction were collected at anthesis, whereas the developmental stage was not a determining factor for DNA isolation.

### 2.2. Alkaloid Extraction and Analysis

Alkaloid extraction and gas chromatography analyses (GC; GC-2014, Shimadzu, Kyoto, Japan) were conducted as described by Kamel et al. [3]. Quantitative and qualitative QA composition was assessed for 29 accessions (Supplementary Table S1). In addition, the GC data for 173 NLL accessions were retrieved from Kamel et al. [3] (Supplementary Table S1). The accessions Brianskij-35 (95826) and Brianskij-123 (95927) were investigated to verify the results obtained by Kamel et al. [3]. Alkaloid abundance was calculated as the mean value from two GC replicates for each accession. Total QA values were estimated as the sum of the major QAs (lupanine, 13-hydroxylupanine, angustifoline, and isolupanine) expressed as a percentage of seed dry weight (SDW). Relative abundance of individual QAs was assessed as the percentage of total QAs (sum of all QAs = 100%).

### 2.3. DNA Isolation

Genomic DNA was isolated from frozen, young leaves using the DNeasy^®^ Plant Mini Kit (Qiagen, Germantown, MD, USA) in accordance with the manufacturer’s protocol. The DNA concentration and quality were determined using a NanoDrop™ ND-1000 spectrophotometer (Thermo Fisher Scientific, Waltham, MA, USA). DNA of six low-alkaloid and six high-alkaloid NLL accessions was extracted for marker development (Table 1). DNA from a total of 202 NLL accessions was isolated to verify consistency between marker segregation pattern and associated seed alkaloid content (Supplementary Table S1).

### 2.4. Amplification and Sequencing

In this study we focused on the *iucundus* candidate gene *RAP2-7* (transcript P27255_008724, corresponding to TanjilG_07628, LOC109342033, and located in scaffold_162_1 within NLL genome assembly v1.0 [25]), which is an AP2/ERF TF previously identified as likely to be a vital gene involved in regulation of QA biosynthesis and accumulation in NLL [13]. 

Six primer pairs covering the entire P27255_008724 transcript sequence as well as the putative promoter sequence (based on the Scaffold 162_1 sequence) were designed. The PCR products were amplified for each primer set in five low-alkaloid (total QA: 0.0005–0.0157% of SDW) and five high-alkaloid (total QA: 1.3809–2.3009% of SDW) NLL accessions, selected from the Polish *Lupinus* Gene Bank collection, and assessed with regard to seed alkaloid content in our previous investigation [3]. For comparative purposes, the DNA sequence of the *RAP2-7* candidate gene and the sequence upstream of the transcribed region, derived from the parental lines (83A:476, low-alkaloid; and P27255, high-alkaloid) of the recombinant inbred lines mapping population were also amplified (GC analysis performed previously by Kroc et al. [13]). The primer sequences and PCR reaction conditions are listed in Supplementary Table S2, and the accessions used for *RAP2-7* DNA amplification are listed in Table 1. Sanger sequencing (using the BigDye^®^ Terminator v.3.1 Cycle Sequencing Kit, Applied Biosystems, Foster City, CA, USA) of each PCR product was performed for all accession. The aligned nucleotide sequences covering both *RAP2-7* transcript and putative promoter were deposited in GenBank (accessions MK834250- MK834264).

Alignment of the genomic sequence of *RAP2-7* for the afore-mentioned six low-alkaloid and six high-alkaloid NLL accessions (Table 1) enabled us to identify conserved polymorphic sites suitable for marker development for MAS in NLL breeding.

In addition, the *RAP2-7* nucleotide sequence was obtained for the three ambiguous accessions, namely Brianskij-35 (95826), Brianskij-123 (95927), and Brianskij-237/83 (95928).

Data on sequence polymorphism in the *RAP2-7* gene and putative promoter sequence were used to compute the Dice similarity coefficients among accessions, which were then utilized in hierarchical clustering (by the average link algorithm) visualized by the dendrogram (Genstat ver. 18, VSN International, Hemel Hempstead, UK).

### 2.5. RNA Isolation and QA Gene Expression Analyses to Confirm Marker Results

Total RNA was isolated from the three NLL accessions investigated in this study, namely 95826, 95927 and 95928. For comparison of gene expression profiles, RNA from six low-alkaloid and five high-alkaloid NLL accessions, previously incorporated in a QA gene expression assay [13], were also used. All accessions used in quantitative PCR (qPCR) analyses are presented in Table 1.

Total RNA was extracted from 30 mg ground leaf tissue using the SV Total RNA Isolation System Kit (Promega, Madison, WI, USA). Reverse transcription was performed with 1 µg RNA using the Transcriptor First Strand cDNA Synthesis Kit (Roche, Mannheim, Germany) following the manufacturer’s procedure. 

The expression profile of the *RAP2-7* candidate gene, as well as three other QA biosynthesis genes previously described for lupins, namely lysine decarboxylase (*LDC*) [10], an acyltransferase-like gene (*LaAT*) [6], and copper amine oxidase (*LaCAO*) [11], were analysed by qPCR. The analyses were run on a LightCycler^®^ 480 Instrument (Roche) using the LightCycler 480 Probes Master, in accordance with the manufacturer’s protocol, in a reaction volume of 10 µL. The primer/probe sequences were designed previously [13] and reaction conditions were optimized for the current experiment. Three biological and two technical replicates of each accession, as well as a negative control, were included in each assay.

PCR amplification efficiencies of the target genes ranged from 0.94 to 0.98, as determined using a standard curve derived from a pooled cDNA mixture. The expression level of the target genes were normalized using three reference genes: α tubulin (*TUBA*), actin 2/7 (*ACT*2/7), and elongation factor 1-β (*ELF*1B), which were selected as the most stably expressed in the current experiment, and chosen from a set of seven candidate genes assessed in our previous work [13]. Gene expression was analyzed using the E-method [26], in relation to NLL accession 96128 (low-alkaloid, *iucundus*) as the calibrator sample. Primers and probes sequences and reaction conditions for the qPCR analyses of selected QA alkaloid genes are presented in Supplementary Table S3.

## 3. Results

### 3.1. Overview of AP2/ERF Transcription Factor DNA Sequence and Development of a Novel, Diagnostic Marker

**Figure 1:**
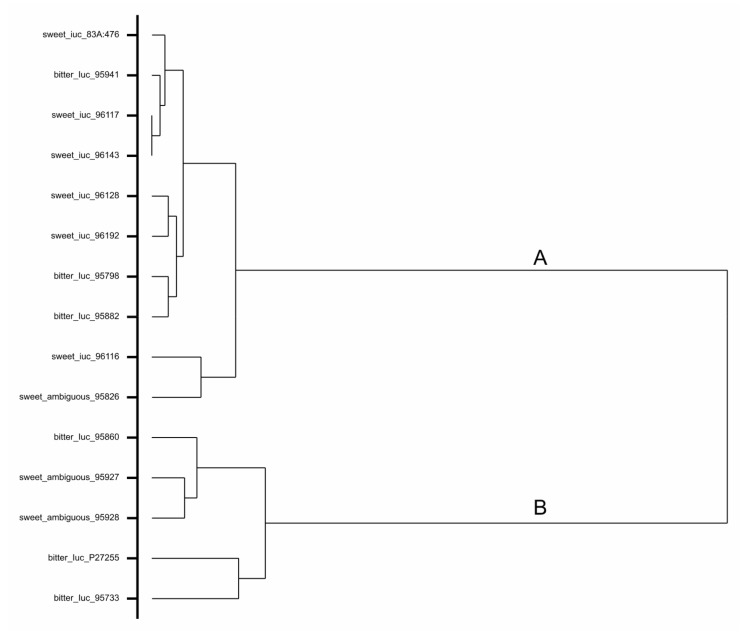
Dendrogram of narrow-leafed lupin accessions based on molecular polymorphism in the *RAP2-7* gene and putative promoter sequence. In hierarchical clustering both sweet (low-alkaloid) *iucundus* and bitter (high-alkaloid) *Iucundus* accessions used in marker development as well as identified upon marker validation three ambiguous accessions characterized with low seed alkaloid content and high-alkaloid marker band were included.

The nucleotide sequence covering a length of ca. 5200 bp was obtained for each of the investigated six low-alkaloid and six high-alkaloid NLL accessions (Table 1). In the amplified sequence, the *RAP2-7* coding region as well as an upstream sequence of ca. 1500 bp were included. Sequence alignment (Supplementary Figure S1) showed that the low-alkaloid accessions differed from high-alkaloid accessions by a total of 84 single-nucleotide polymorphisms (SNPs) and small insertions/deletions (INDELs) (1–12 bp). The low-alkaloid accessions were highly similar and varied only in a relatively small number of SNPs and small INDELs (Supplementary Figure S1). This is illustrated by the fact that all of them belonged to cluster A, one of the two main clusters on a dendrogram (Figure 1). Among the high-alkaloid accessions, two groups sharing the majority of SNPs and small INDELs could be distinguished (Supplementary Figure S1). The first group comprised the accessions Bitter Blaue Lupine (95798), Population-29b (95882), and Azuro (95941) belonging to cluster A (Figure 1), while the second group included the accessions Petakli Tikwa (95860), Population B-530/79 (95733), and the mapping population paternal line P27255 belonging to cluster B (Figure 1).

**Figure 2:**
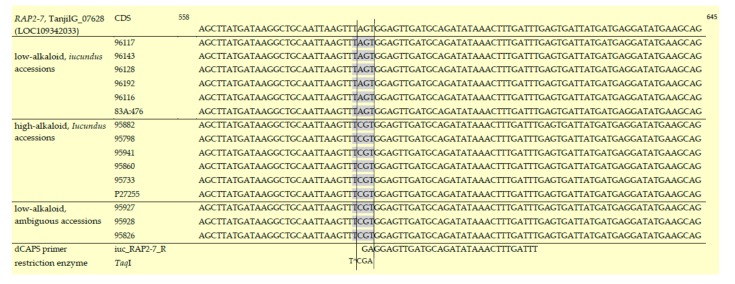
Alignment of *RAP2-7* transcription factor DNA sequences (exon 4) from low-alkaloid and high-alkaloid narrow-leafed lupin accessions. The key mutation (AGT\CGT) used in iuc_RAP2-7 marker development is shown together with the reverse derived cleaved amplified polymorphic sequence (dCAPS) primer (complement sequence) introducing the TaqI restriction site (indicated by gray shading).

The alignment of *RAP2-7* DNA sequences also revealed that the nonsynonymous substitution of amino acid Serine into Arginine (AGT\CGT, S196R, numbering according to the XP_019435537.1 RefSeq protein for locus TanjilG_07628 [25]) was the only polymorphic site within the coding sequence (exon 4) that was conserved in all high-alkaloid vs. low-alkaloid accessions (Table 1, Figure 2, Supplementary Figure S1), and was associated with the estimated total alkaloid content in seeds. Therefore, this mutation was further used in marker development. The *RAP2-7* genomic sequences directly upstream from the transcription initiation codon did not contain any polymorphic sites that clearly differentiated the sweet and bitter genotypes. 

The identified SNP did not create a recognition site for any restriction endonuclease, therefore we developed a derived cleaved amplified polymorphic sequence (dCAPS) by introducing one mismatch into the reverse primer sequence [27]. This dCAPS marker was designated iuc_RAP2-7. A pair of primers, iuc_RAP2-7_F (5′-TCGGAACCTATTTAAGTGGCTG-3′) and iuc_RAP2-7_R (5′-AAATCAAAGTTTATATCTGCATCAACTCCTC-3′), were designed to amplify a PCR product of the size 258 bp. The introduced polymorphism, together with the single nucleotide mutation occurring in the DNA sequence, created a cleavage site recognized by the TaqI restriction enzyme (Figure 2). The developed iuc_RAP2-7 marker was characterized by a high-alkaloid marker band of length 226 bp (apart from a 32 bp cut-off product, not visible in the agarose gel) and a low-alkaloid marker band 258 bp in length (Supplementary Figure S2). Primer sequences and the PCR conditions are presented in Supplementary Table S2.

### 3.2. Variation of Seed QA Content among Representatives of NLL Collection, Incorporated in Marker Validation

**Figure 3:**
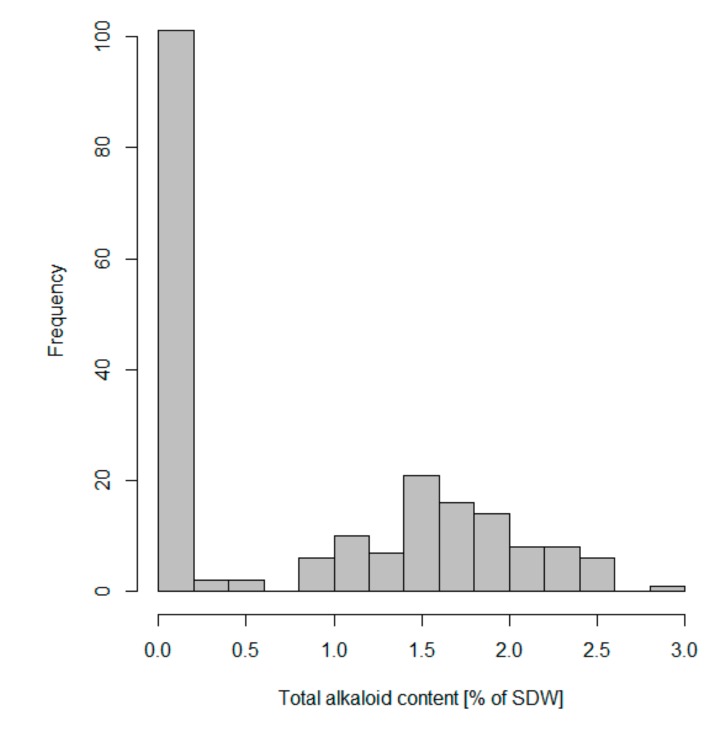
A histogram showing a bimodal distribution of total alkaloid content in 202 narrow-leafed lupin accessions used in iuc_RAP2-7 marker validation.

Among the set of 202 NLL accessions used for marker validation, total alkaloid content varied from 0.0005% to 2.8752% of the SDW (Supplementary Table S1). The distribution of total alkaloid content was found to be bimodal, what supports the presence of a major gene regulating seed alkaloid content (Figure 3). The collection comprised 56 accessions with distinctly low seed alkaloid content, which did not exceed the accepted industry limit of 0.02% of the SDW, as well as 146 accessions that exceeded this limit (Supplementary Table S1, total QA values rounded to two decimal places). As an alkaloid content above the accepted threshold is not equatable to a bitter phenotype, for the purpose of further investigation we distinguished 97 accessions with distinctly high-alkaloid content ≥0.9% of the SDW, and 49 accessions with intermediate alkaloid content (>0.02% and ≤0.5% of the SDW) (Supplementary Table S1). In the assessed material no accessions with total alkaloid content between 0.5% and 0.9% of the SDW were identified.

Considering total alkaloid content across the classes of origin, the lowest mean of 0.27% of the SDW was observed in the CV category, however only 33 out of 76 genotypes within this group were characterised with distinctly low-alkaloid content (≤ 0.02% of the SDW) (Supplementary Table S1, total QA values rounded to two decimal places). The highest mean total alkaloid content (1.59% of the SDW) was recorded for the CO class represented mostly by accessions with alkaloid content above accepted industry limit (59 out of 60) (Supplementary Table S1). The XD class, representing improved accessions, was the most diverse regarding total alkaloid content (17 accessions ≤ 0.02% of the SDW, 26 accessions > 0.9% of the SDW, and 14 accessions with intermediate alkaloid content) (Supplementary Table S1).

### 3.3. iuc_RAP2-7 Marker Validation in NLL Collection

The developed iuc_RAP2-7 dCAPS marker was amplified in 202 accessions gathered in the Polish *Lupinus* Gene Bank, encompassing different classes of origin. In the observed digestion pattern a low-alkaloid marker band was scored as 1, whereas a high-alkaloid marker band was scored as 2 (Supplementary Table S2).

In the investigated material, the accessions characterized with an alkaloid content up to 0.5% of the SDW exhibited a low-alkaloid marker band and the underlying SNP mutation, whereas in the case of accessions with seed alkaloid content ≥0.9% of the SDW we identified a high-alkaloid marker band and associated SNP (Supplementary Table S1, Supplementary Figure S2).

In the case of three accessions, namely Brianskij-35 (95826, XD), Brianskij-123 (95927, CV), and Brianskij-237/83 (95928, XD), in which the seed alkaloid content was assessed by GC analysis as lower than 0.5% of the SDW (ranging from 0.0851% to 0.1895% of the SDW), we unexpectedly identified a bitter-allele marker band (Supplementary Table S1, Supplementary Figure S2). These three accessions originated from the All-Russian Lupin Institute, Bryansk, Russia.

To confirm these results for the ambiguous accessions, new seed samples of two available accessions (Brianskij-35 and Brianskij-123) were obtained from a different source. The remaining accession Brianskij-237/83 was inaccessible in any other national or international gene bank. The acquired seeds were used for both GC analysis (Table 1, Supplementary Table S1) and a greenhouse experiment for subsequent DNA extraction and marker re-evaluation (Supplementary Table S1). Both accessions were confirmed to show low-alkaloid contents, and again demonstrated the allele band characteristic of high-alkaloid accessions (Table 1, Supplementary Table S1, Supplementary Figure S2).

### 3.4. Assessment of DNA Sequence of Ambiguous Accessions

The AP2/ERF TF DNA sequences obtained for Brianskij-237/83, Brianskij-35, and Brianskij-123 were aligned with sequences for the six low-alkaloid and six high-alkaloid accessions used in iuc_RAP2-7 marker development. The three ambiguous accessions showed, in general, much higher sequence similarity to high-alkaloid accessions, including the key mutation used in iuc_RAP2-7 marker development, which was here characteristic for the *Iucundus* accessions (Figure 2). In more detailed comparison, the *RAP2-7* sequences of Brianskij-123 (95927) and Brianskij-237/83 (95928) were most similar to the subgroup encompassing bitter accessions: Petakli Tikwa (95860), Population B-530/79 (95733), and P27255 parental line (Figure 1, cluster B). The Brianskij-35 (95826) accession shared high sequence similarity to Bitter Blaue Lupine (95798), Population-29b (95882), and Azuro (95941) (Figure 1, cluster A). No polymorphic sites unique to the three ambiguous accessions, when compared with the bitter *Iucundus* forms, were identified (Supplementary Figure S1).

### 3.5. RAP2-7, LDC, LaAT, and LaCAO Gene Expression Analysis

**Figure 4:**
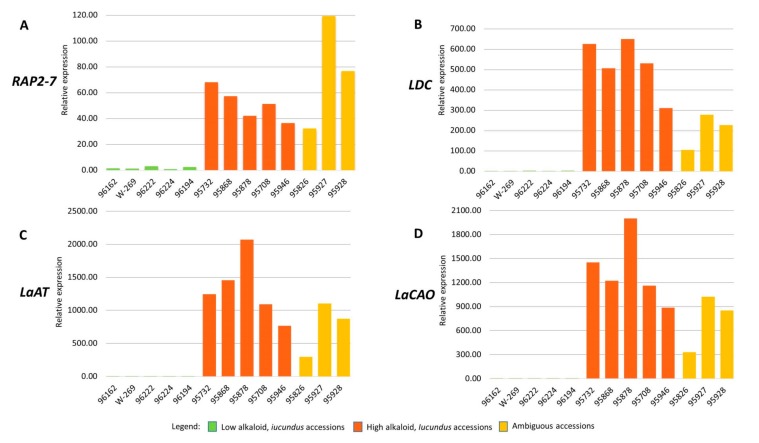
Expression profiles of four lupin alkaloid genes (**A**–**D**) assessed by quantitative PCR analysis. Bars indicate fold-change expression in relation to the calibrator (96128, low-alkaloid, *iucundus* accession). Relative quantification was normalized to three reference genes (α tubulin, actin 2/7, and elongation factor 1-β). (**A**) APETALA2/ethylene response transcription factor, *RAP2-7*, (**B**) lysine decarboxylase (*LDC*), (**C**) acyltransferase-like gene (*LaAT*), (**D**) copper amine oxidase (*LaCAO*).

Previously, we demonstrated that, based on comparative transcriptomic analysis as well as further qPCR validation, *RAP2-7* showed significant differential expression (*P* < 0.0001) in high-alkaloid (*Iucundus*) vs low-alkaloid (*iucundus*) NLL accessions [13]. The higher expression level in *Iucundus* forms was confirmed for three additional QA genes [13]. In the current investigation, we analysed the expression levels of *RAP2-7*, *LDC*, *LaAT*, and *LaCAO* in the three ambiguous NLL accessions: Brianskij-35 (95826), Brianskij-123 (95927), and Brianskij-237/83 (95928). For comparative purposes, six *iucundus* and five *Iucundus* NLL accessions that were previously analyzed [13] were also incorporated in the qPCR assays. The list of all accessions analyzed is shown in Table 1. The relative expression level of *RAP2-7* in the three ambiguous NLL accessions was much higher than that in *iucundus* accessions, when assessed in relation to 96128 (*iucundus*) as a calibrator. The expression of *RAP2-7* in lines 95927 and 95928 was higher than that in the assessed *Iucundus* accessions (Figure 4A). The expression level of the other three QA genes in the ambiguous accessions also exceeded the level detected in *iucundus* lines and was comparable to that observed in *Iucundus* forms (Figure 4B–D).

## 4. Discussion

High content of seed alkaloids diminishes the food and feed value of lupins, therefore reduction of the alkaloid content remains an important objective in both lupin breeding and supporting research. To date, intensive breeding efforts have been dedicated to decrease seed QA content in NLL. For example, the Polish *Lupinus* Gene Bank collection includes accessions with total QA content of 0.0005% of the SDW [3], which is notably lower than that of existing NLL cultivars as well as the accepted industry threshold [2,3]. Further crop improvement, regarding low QA phenotypes, might be greatly facilitated by the establishment and exploitation of the genotype–phenotype relationship through genomics-assisted breeding.

Previously, we described a transcriptome-derived candidate gene, *RAP2-7* TF, that co-segregates with the *iucundus* locus and is likely to be a crucial gene involved in regulation of QA biosynthesis and accumulation in NLL [13]. In the present investigation, we exploited the point mutation in the fourth exon of *RAP2-7* TF associated with alkaloid content, to develop a co-dominant gene-targeted marker useful in MAS. Owing to its co-dominant segregation, the proposed iuc_RAP2-7 marker is suitable to distinguish homozygous and heterozygous NLL plants, which would not be possible through any standard method simply assessing seed alkaloid content. SNP markers have been extensively used in molecular breeding of many plant species because they represent the most abundant class of polymorphisms distributed throughout plant genomes, and are considered to play major roles in triggering phenotypic variation [18,28]. While mutations are random, the driving force is selection, which shapes the observable protein range by favoring those mutations that maintain or improve a phenotype [29]. Amino acid substitution, a type of genetic point mutation, is one basic event that can drive evolution [30]. It may have an effect on protein three-dimensional structure and consequently on protein function [29]. The proposed marker was anchored in *RAP2-7* gene sequence, which is possibly a key regulator of QA biosynthesis/accumulation in NLL [13]. Therefore, the iuc_RAP2-7 marker is potentially of functional relevance. In case of functional markers, causally affecting phenotypic trait variation there is no risk of undesirable recombination in the chromosome region, which may occur if genetic intervals exist between a marker and a gene [31]. With regard to markers linked to a gene/trait of interest, such recombination during evolution or in the plant breeding process may result in the accessions exhibiting the desirable marker genotype, but not necessarily harboring the target alleles, giving rise to “false positive” results and therefore limiting marker utility [31]. Presented marker-trait association in the case of iuc_RAP2-7 marker can be of great support to define its relevance and validity. Nonetheless, bearing in mind that *RAP2-7* candidate gene has not been functionally validated yet, segregation between iuc_RAP2-7 marker and *iucundus* locus cannot be excluded.

As a result of lupin alkaloids toxicity, only very low quantities of these compounds are permitted in fodder and food. To meet the demands of farmers and consumers, a limit of 0.02% of the SDW has been established as an acceptable safe, low-alkaloid threshold. This standard limit is, however, only contractual and the exact distinction in the alkaloid content associated with the sweet genotype, and the threshold that, if exceeded, leads to the bitter genotype, has not been defined yet. We observed complete agreement between the iuc_RAP2-7 high-alkaloid marker band and phenotype data for 97 NLL accessions with seed alkaloid content ≥0.9% of the SDW. In the case of 102 accessions characterized by seed alkaloid content up to 0.5% of the SDW, a low-alkaloid marker band and the underlying SNP mutation were observed. The exact threshold value that would be detectable with the proposed marker is, however, still difficult to define because no accessions with total alkaloid content between 0.5% and 0.9% of the SDW were included among the assessed material, and are not common in general, as a result of selection pressure towards significant reduction of alkaloid content in NLL seeds. Further determination of precise iuc_RAP2-7 marker cut-off value would be desirable. Moreover, given that within the sweet genotypes large variation in seed alkaloid content exist, it is apparent that along with the major gene, additional genes should be involved in shaping the observed phenotypic diversity. Our previous results showed, that a broad-sense heritability for total alkaloid content in NLL mapping population of RILs was 91.15% (phenotypic data collected across five years), what suggests a high level of genetic control for this trait [13]. Nonetheless, alkaloid content in lupin seeds is also known to be affected by environmental factors [32,33]. Taken together, our results provide new insight into a complex picture of QA accumulation in NLL seeds, however further investigation is necessary to fully recognize all factors capable of modulating alkaloid accumulation in accessions with distinctly low alkaloid content in seeds.

In lupin breeding programs, wild accessions are often used in crosses to broaden the gene pool of domesticated germplasm [16]. Early and efficient selection of NLL genotypes with low seed alkaloid content is currently hampered by the fact that reliable phenotypic selection requires post-harvest seed chromatographic analyses, which precludes rapid progress in development of new cultivars and contributes to the associated cost. Detection of a DNA marker diagnostic for superior alleles of a gene that controls natural variation in a desirable agronomic trait, at early steps of the breeding process, might greatly reduce breeding program cycles and the number of genotypes required to be evaluated. Therefore, the use of such DNA-based diagnostic markers has proved to be both time and cost efficient in plant breeding [34,35]. The iuc_RAP2-7 DNA marker described here meets important criteria for use in MAS, i.e., high reliability, standard DNA quantity and quality requirements, procedural simplicity, accurate genotype discrimination, and low cost [36]. Such a breeder-friendly and predictive molecular marker could be directly deployed in lupin breeding programs. 

At present, NLL has been the focus of the most advanced achievements in research targeted at unraveling alkaloid biosynthesis in lupins, as well as in selection of low-alkaloid cultivars through breeding efforts. Among several single recessive genes associated with reduced alkaloid content, the *iucundus* allele has been incorporated in the majority of NLL cultivars [15]. Intercrossing of genotypes harboring different low-alkaloid-content genes might lead to the bitter phenotype owing to gene complementation [16]. Selection of a specific genotype for breeding purposes should therefore not be guided by its low total alkaloid content only, as there is no guarantee which QA gene regulating alkaloid synthesis is in action. To avoid contamination in NLL collections it is desirable to develop databases that monitor gathered accessions with regard to possession of genes that confer low-alkaloid contents. Within the present set of investigated NLL plant material we encountered three accessions (Brianskij-35, Brianskij-123, and Brianskij-237/83) assessed as low-alkaloid by GC seed analysis, but characterized by a *RAP2-7* DNA sequence of high similarity to that of bitter accessions, which included the key mutation incorporated in the iuc_RAP2-7 marker and generated a marker band characteristic of high-alkaloid accessions. The expression level of *RAP2-7* and three additional NLL QA genes, namely *LDC*, *LaAT*, and *LaCAO*, were in the leaves of the Brianskij accessions comparable to that in high-alkaloid *Iucundus* accessions (Figure 4). Given that the four genes were mapped across different linkage groups [13] and similar expression profiles were observed in the three ambiguous accessions, it is likely that, in these accessions, a different gene is in action that regulates alkaloid biosynthesis/accumulation. It is also possible that the low seed QA content in these accessions arises from blocked transport of alkaloids, which are synthesized in the leaves. In this respect, it would be worthwhile to determine whether the low seed alkaloid content in these ambiguous accessions corresponds to the content in leaves and other aerial tissues. Thus, the Brianskij accessions are highly interesting research material, as an apparently novel source of mutation that confer reduced alkaloid content in the seeds, or potentially a long-sought-after form characterized by high QA content in the leaves combined with sweet seeds. Such a phenotype is considered to be extremely beneficial given that lupin alkaloids play an important role in plant chemical defense against herbivores and pathogens [1]. Further investigation of these accessions might considerably improve our understanding of QA alkaloid biosynthesis and its regulation in NLL.

Considering that the three Brianskij accessions likely represent a novel source of sweetness, we can conclude that in the case of the remaining 199 NLL accessions possessing the sweet *iucundus* or bitter *Iucundus* mutation, the overall matching rate of the iuc_RAP2-7 marker was 100%, given that the detection limit is 0.5% of the SDW. This material comprised both domesticated and wild germplasm. From a consumption perspective, the iuc_RAP2-7 marker provides a promising tool for high-throughput rejection of distinctly bitter *Iucundus* genotypes (≥0.9% of the SDW). However, it would not allow one-step, rapid selection of accessions with a safe seed alkaloid content for feeding purposes (below 0.02% of the SDW). The iuc_RAP2-7 marker bands detected for the Brianskij and *Iucundus* accessions had the same size on agarose gel, indicating that the proposed marker is not capable of discriminating which low-alkaloid gene is in operation in a certain accession. Nonetheless, the observed banding pattern appears to be advantageous from the perspective of its exploitation in breeding. The reason for this is that accessions such as the Brianskij genotypes would be unconsciously discarded together with bitter *Iucundus* lines during selection, thereby promoting utilization of the *iucundus* allele to maintain pure-breeding, low-alkaloid cultivars. Therefore, although the proposed marker does not provide the opportunity for easy selection of extremely sweet genotypes, it also will not lead to the introduction of high-alkaloid accessions as “false positive” selection results in the breeding process, and thus does not pose a risk for potential bitter seed contamination.

To conclude, we demonstrated that the iuc_RAP2-7 marker offers a powerful tool for improvement in the efficiency of the selection process and development of improved low-alkaloid genotypes of NLL in conventional breeding programs. The outcome of this study will therefore promote selection of superior seed quality in NLL.

## Figures and Tables

**Table 1:** List of accessions used in APETALA2/ethylene response transcription factor (*RAP2-7)* DNA sequence assessment for iuc_RAP2-7 marker development, as well as quantitative PCR (qPCR) analyses. For each accession total and individual quinolizidine alkaloid content is presented (from previous or present gas chromatographic [GC] analyses).

Catalogue No. ^a^	Accession Name	Total Alkaloid Content (% of Seed Dry Weight)	Lupanine (%)	13-Hydroxylupanine (%)	Angustifoline (%)	Isolupanine (%)	GC Analysis	*RAP2-7* DNA Sequence Assessment and Marker Development	qPCR Analysis
low-alkaloid accessions									
96143	Danja ^b^	0.0022	45.45	40.91	13.64	-	Kamel et al. (2016)	+	
96117	Yandee ^b^	0.0029	31.03	48.28	13.79	6.90		+	
96116	IIIyarie ^b^	0.0032	31.25	50.00	12.50	6.25		+	
96192	Bordako ^b^	0.0157	52.87	33.76	7.64	5.73		+	
–	83A:476, DxW maternal line ^b^	0.0829	65.36	21.34	11.65	1.65	Kroc et al. (2019)	+	
96128	Stadolishchienskij L-569 ^b^	0.0004	22.26	60.86	16.81	-		+	+
–	W-269 ^b^	0.0010	46.31	43.89	4.06	5.75			+
96162	Gunguru ^b^	0.0127	50.23	37.83	9.95	2.00			+
96224	W-226B ^b^	0.0146	69.64	26.31	1.49	2.56			+
96222	W-197 ^b^	0.0250	71.02	21.94	1.80	5.25			+
96194	Borweta ^b^	0.0299	73.06	17.58	5.10	4.25			+
95826	Brianskij-35 ^b^	0.1205	31.78	37.18	15.52	15.52	Kamel et al. (2016)		
	Brianskij-35 ^c^	0.0851	38.16	26.09	19.81	15.93	present study		+
95927	Brianskij-123 ^b^	0.1895	24.22	41.85	19.53	14.41	Kamel et al. (2016)		
	Brianskij-123 ^c^	0.1160	42.03	20.55	20.06	17.36	present study		+
95928	Brianskij-237/83 ^b^	0.1200	28.67	38.67	17.17	15.50	Kamel et al. (2016)		+
high-alkaloid accessions									
95941	Azuro ^b^	1.3809	56.24	26.73	15.55	1.48	Kamel et al. (2016)	+	
95798	Bitter Blaue Lupine ^b^	1.8431	52.46	27.94	18.22	1.37		+	
95882	Population-29 b	2.1170	46.09	30.71	20.67	2.52		+	
95860	Petakli Tikwa ^b^	2.2960	21.41	50.71	27.52	0.36		+	
95733	Population B-530/7 ^b^	2.3009	56.44	25.23	18.16	0.17		+	
–	P27255, DxW paternal line ^b^	2.7272	67.75	16.40	13.69	2.16	Kroc et al. (2019)	+	
95946	Morsico Pop.1100 ^b^	2.4305	43.07	33.96	22.80	0.17			+
95878	Population-4 ^b^	2.4975	12.76	62.16	24.81	0.26			+
95868	Population-22746 ^b^	2.5024	9.82	65.03	24.90	0.25			+
95732	Population B-529/79 ^b^	2.5124	58.52	25.68	15.57	0.19			+
95708	Badajoz-3 ^b^	2.8751	13.63	66.45	19.64	0.29			+

^a^ Polish *Lupinus* Gene Bank; ^b^ Seeds source: Poznan Plant Breeders Ltd., Wiatrowo Branch, Poland.; ^c^ Seeds source: National Centre for Plant Genetic Resources: Polish Genebank, Radzików, Poland.

## Supplementary Materials

The following are available online at https://www.mdpi.com/2073-4425/10/6/428/s1, **Figure S1:** Graphic representation of the polymorphic sites identified in *RAP2-7* gene and putative promoter sequence (visualized in Geneious software), within a set of low-alkaloid and high-alkaloid NLL accessions. mRNA and coding sequence were included for comparison. The consensus sequence is shown at the top. Bars represent the sequences. Base pairs that are identical to the consensus are shown in gray, SNPs are shown as vertical colored lines (red = A, blue = C, orange = G, and green = T), whereas deletions are represented as gaps, **Figure S2:** Validation of the iuc_RAP2-7 dCAPS marker in selected low-alkaloid and high-alkaloid narrow-leafed lupin accessions from the Polish *Lupinus* Gene Bank collection. Total alkaloid content measured as a percentage of the seed dry weight is shown in parentheses. The marker was resolved in 2% agarose gel against a size ladder presented on the left side (L). The accession order is as follows: (1) 96128, Stadolishchienskij L-569 (0.0005); (2) 96143, Danja (0.0022); (3) 96117, Yandee (0.0029); (4) 96250, Wars (0.0027); (5) 96116, IIIyarie (0.0032); (6) 96217, Borlu (0.0086); (7) 96188T, Reduced Branching (0.0201); (8) 96169, R85L_460_Kalya (0.0231); (9) 95916T, BRGC-10271 (0.3596); (10) 96110, Ignis (0.4494); (11) 96377, LIT 11-143 (0.5019); (12) 96108, Obornicki (0.8989); (13) 95932, Population-25097 (0.9164); (14) 95711, Badajoz-4 (0.9688); (15) 95716, Hinojoso de Duero-7 (0.9741); (16) 95848, Population-22876 (1.0089); (17) 95799, Palestyna (1.5977); (18) 95746, Population B-552/79b (1.8386); (19) 95733, Population B-530/79 (2.300); (20) 95868, Population-22746 (2.5024); (21) 95706, Vitigudino-1 (2.5433); (22) 95708, Badajoz-3 (2.8752); (23) 83:476 DxW maternal line (0.0829); (24) P27255 DxW paternal line (2.7272); (25) 95928, Brianskij-237/83 (0.12); (26) 95826, Brianskij-35b (0.1205); (27) 95927, Brianskij-123 (0.1895), **Table S1:** List of NLL accessions used in iuc_RAP2-7 marker validation, together with their total and individual quinolizidine alkaloid content and marker evaluation results, **Table S2:** Primer sequences and PCR reaction conditions for the amplification of *RAP2-7*, **Table S3:** Primer/probe sequences and reaction conditions for the quantitative PCR analyses of selected quinolizidine alkaloid genes.
