# Supplementary material for: Development and Validation of a Gene-Targeted dCAPS Marker for Marker-Assisted Selection of Low-Alkaloid Content in Seeds of Narrow-Leafed Lupin (Lupinus angustifolius L.)
Source: Genes (Basel). 2019 Jun 4;10(6):428. doi: 10.3390/genes10060428 (PMC6628303; doi:10.3390/genes10060428)
Supplement: Supplementary file 1 [file genes-10-00428-s001.zip › Table S2_Table S3.pdf]

Table S2. Primer sequences and PCR reaction conditions for the amplification of *RAP2-7*.

Sequence	Primer name	Primer sequence	Product lenght	Tm	Elongation time	Mg
RAP2-7 transcript	RAP_Lang_F1	TCAATCCAAGTCCTTTTGTCC	610 bp	55	45 s	2 μM
	RAP_Lang_R1	CAGGTTCAACCATTCAGTTTTT				
	RAP_Lang_F2	AGAGTTTCTGATATGAACAAGAG	2076 bp	50	60 s	2 μM
	RAP_Lang_R2	TAGGCTCAAATCAAGATTATGAC				
	RAP_Lang_F3	TGTTAGTAGTTTTATTTTAGGTGA	1387 bp	55	60 s	2 μM
	RAP_Lang_R3	GTACGATTTCATATAAAACATGAC				
putative promoter	RAP_Lang_F4	GAGAAGAGAAATAGTTGGATG	561 bp	55	60 s	2 μM
	RAP_Lang_R4	CAGAAAAAGAGAAGAATAGAAAC				
	RAP_Lang_F5	TTACTAAACGGTTCATAGACT	510 bp	55	60 s	2 μM
	RAP_Lang_R5	CATCAATCCAAGATATTCACT				
	RAP_Lang_F6	TTGGCACTCTAACGCAAC	676 bp	53	60 s	2,5 μM
	RAP_Lang_R6	ATGTTCTATTTCTTTTGATTTTTC				
Iuc_RAP2-7 marker	RAP_dCAPs_F	TCGGAACCTATTTAAGTGGCTG	258 pz	59	30 s	2 μM
	RAP_dCAPs_TaqI_R	AAATCAAAGTTTATATCTGCATCAACTCCTC				

Table S3. Primer/probe sequences and reaction conditions for the quantitative PCR analyses of selected quinolizidine alkaloid genes.

Target gene	Locus name in NLL genome /NCBI GeneID	PCR thermal profile (°C)	qPCR reaction mixture[b]			Primer sequence[a]	Probe sequence[a]	Product length (bp)	Efficiency
			Primer pairs (µM)	Probes (µM)	Template cDNA (µl/reaction dilution)				
LaAT	TanjilG_21586 /LOC109328823	58	0.5	0.1	1 /1:10	F: CCACCTTCCAAAGCCTTATT<br>R: GTGTTGCCATGCCTAAGTTT	TAACCTTCAGAGCTTCCATCTCCTCTC	146	0.94
LaCAO	TanjilG_00530 /LOC109328478	60	0.5	0.1	1 /1:10	F: TCCTAATCAAAATCCACGCATTG<br>R: GTGACTCCAAATACATACCAAAG	CCTACATGGGTTACGCAGAACCGATCT	105	0.97
LDC	TanjilG_09726 /LOC109327937	58	0.5	0.1	1 /1:10	F: ATTGGTGGCGGTTTCACTTG<br>R: AAAGGTGACTCAGCAAAATAACG	TGGAAAAGAGGAAGGTGTTGTGGTAATTGGA	140	0.98
RAP2-7	TanjilG_07628 /LOC109342033	58	0.8	0.1	1 /1:10	F: TATCACATTCTGGGGCATCC<br>R: CCACTCCCTTGTTCCTTTCA	TCCTGTGGAGGAAGATTCCTCAAACAA	135	0.95

[a] Kroc et al. (2019)

[b] total reaction volume 10 (µl)
