# Supplementary figures and images for: Development and Validation of a Gene-Targeted dCAPS Marker for Marker-Assisted Selection of Low-Alkaloid Content in Seeds of Narrow-Leafed Lupin (Lupinus angustifolius L.)
Source: Genes (Basel). 2019 Jun 4;10(6):428. doi: 10.3390/genes10060428 (PMC6628303; doi:10.3390/genes10060428)

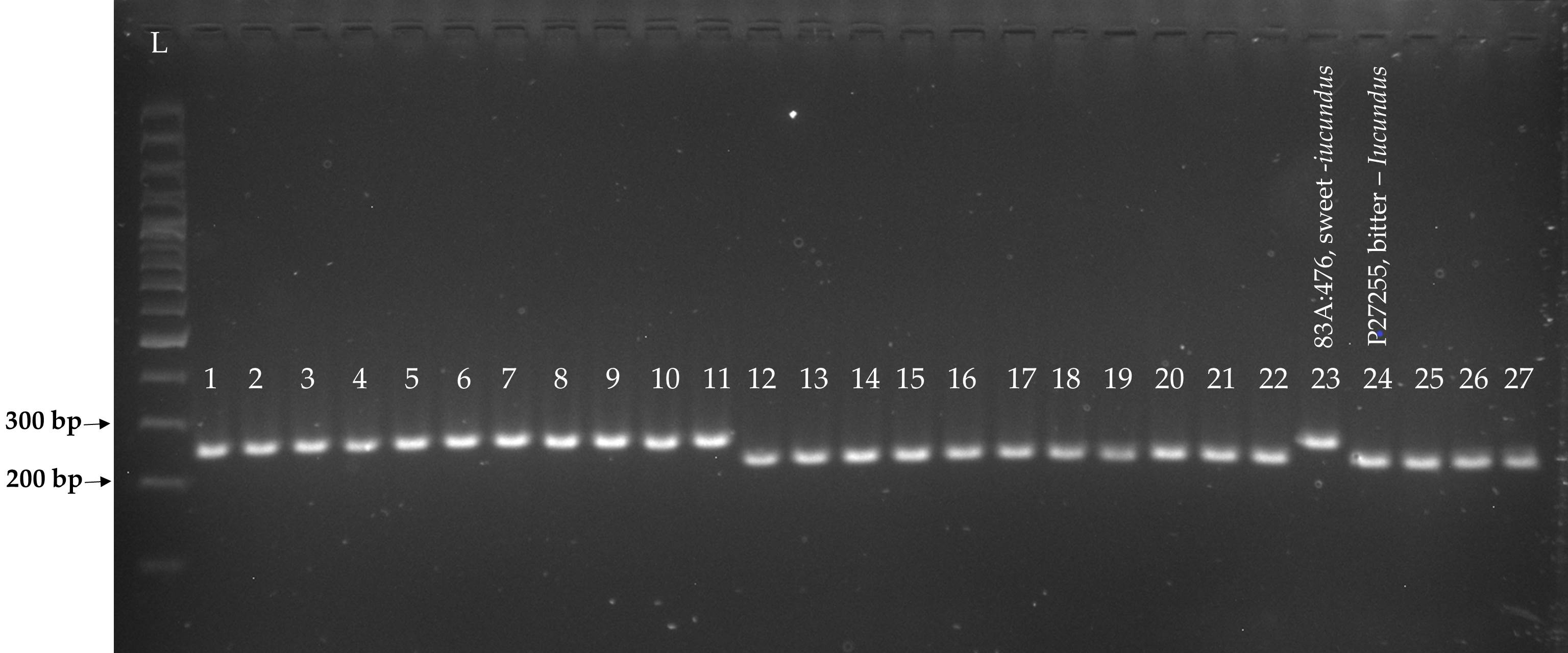

Supplement: Supplementary file 1 [file genes-10-00428-s001.zip › Figure S2.jpg]

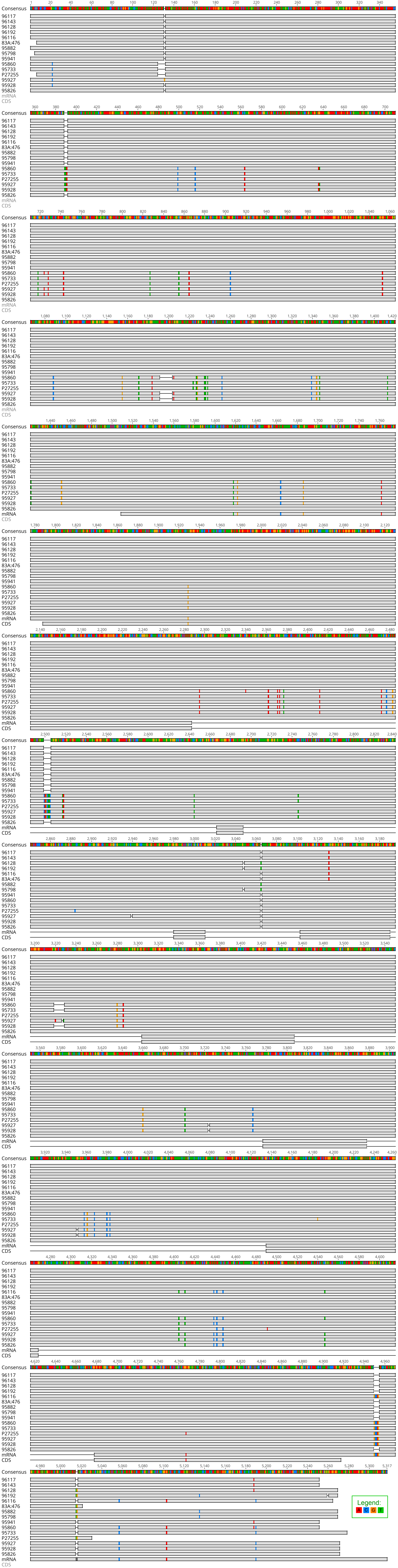

Supplement: Supplementary file 1 [file genes-10-00428-s001.zip › Figure_S1.pdf]
